# The Carcinogenesis of the Human Scalp: An Immunometabolic-Centered View

**DOI:** 10.3390/ijms252212064

**Published:** 2024-11-10

**Authors:** Baruch Kaplan, Rebecca von Dannecker, Jack L. Arbiser

**Affiliations:** 1 Adelson School of Medicine, Ariel University, Ariel 40700, Israel; drbakaplan@gmail.com; 2 Marilia Medical School, Marilia 17519-030, SP, Brazil; rebeccavondannecker@gmail.com; 3 Metroderm/United Dermatology Partners, 875 Johnson Ferry RD, Atlanta, GA 30342, USA

**Keywords:** aging, stroma, Sirt3, ultraviolet light, IRF4

## Abstract

The human scalp is a common site of skin cancer in humans, with nonmelanoma skin cancer being exceedingly common. In this review, two dermatologists with extensive experience in cutaneous oncology will discuss unique features of the epidemiology of cancer of the scalp. Clinical observations on these common skin cancers lead to insight into the pathogenesis and potential prevention and treatment of cutaneous scalp neoplasia. Our hypothesis is that the presence of hair protects against the development of skin cancer but not by serving as a physical shield but rather by providing continuous IL-17-biased immunosurveillance. The loss of hair allows for a release from immunosurveillance, resulting in the expansion of neoplastic cells towards skin cancer. Both hair follicles and metabolic changes in stroma allow for permissiveness for tumor promotion.

## 1. Introduction

The scalp is an exceedingly common site for basal cell and squamous cell carcinoma and occasional melanomas. Benign lesions, including seborrheic keratoses and blue nevi, are also commonly found on the scalp. A major difference between the scalp and other common sun-exposed areas in terms of skin cancer risk is that the scalp is covered with hair during periods of peak sun exposure during youth. Carcinomas on the scalp are more common in men than women, but they may become evident in women with sparse hair. Androgenetic alopecia is also common in men, beginning in the 20–30-year-old age group and becoming more evident with age. Women too lose hair but not usually to the same extent as men. The incidence of scalp carcinomas is also higher in men than in women [1,2]. Androgenetic alopecia is thought to be mediated by dihydrotestosterone, and this has led to the use of dihydrotestosterone blockers as a common treatment for alopecia [3].

Two major differences exist in the scalp of elderly men vs. elderly women. The first is the presence of hair. While elderly women experience hair thinning, frank alopecia is uncommon. The second change is that of the stroma. Stromal changes may be responsible for the miniaturization of hair follicles, and stromal changes associated with miniaturization may also favor the development of actinic keratosis (AK) and nonmelanoma skin cancer (NMSC) [3,4].

Scalp stroma is subject to aging as is every other tissue. A candidate for aging in scalp stroma is Sirt3, a mitochondrial deacetylase which stimulates mitochondrial biogenesis [5,6]. TGF-beta lowers Sirt3 levels, promotes fibrosis, and is decreased in fibrotic diseases such as scleroderma. Of interest, the scleroderma dermis does not support hair growth, and a similar mechanism of fibrotic stroma may both decrease hair growth and favor the development of cutaneous neoplasia [7,8].

## 2. Hair Cycle and Aging

The hair growth cycle consists of three main phases: anagen, catagen, and telogen. Throughout life, hair follicles undergo repeated cycles of growth and rest, which involve changes in the morphology and structure of the dermal papilla (DP), the formation of new hair shafts (HSs), and the shedding of old hair. The anagen phase is the active growth period, during which rapid hair growth and complete HS formation occur. This phase begins when quiescent signals from the inner bulge layer and other hair follicle stem cell (HFSC) niches are overridden by a combination of BMP inhibitory and Wnt activation signals. The anagen phase can last between 2 and 6 years, depending on genetic factors. Following this, the catagen phase, lasting about 2 to 3 weeks, serves as a brief transitional stage marked by follicular regression, where the apoptosis of hair bulb cells leads to degeneration. During this time, HS growth halts, cell proliferation and differentiation decline, and the DP shrinks. The telogen phase is the resting period, during which proliferative and biochemical activity levels reach their lowest point. The hair, now referred to as “club hair,” separates from the DP and is shed. This phase typically lasts 2 to 3 months, after which DP cells migrate to the bulge, activating stem cells and initiating the next anagen phase [9].

With aging, the hair cycle undergoes significant changes, resulting in a gradual reduction in hair density, thickness, and pigmentation. The anagen phase shortens, leading to thinner HSs, while the interval between hair shedding during telogen and the onset of new growth in anagen lengthens. HFSCs, crucial for hair regeneration, enter a prolonged quiescent state as aging advances. This quiescence, along with changes in the follicle’s extracellular environment, leads to the miniaturization of hair follicles, resulting in thinner and less dense hair. Molecular signaling disruptions, such as increased BMP signaling and decreased Wnt activation, further impair hair regeneration. These accumulated imbalances in cellular signaling compromise HFSC function, resulting in diminished hair production; the emergence of thinner, graying hair; and ultimately, progressive hair loss [3,10].

## 3. Actinic Keratosis and Cutaneous Squamous Cell Carcinoma

AK and cSCC are exceedingly common lesions that occur on the scalp. AK is a known precursor to cSCC, with a small but significant number of AK cases progressing to cSCC. For this reason, the destruction of AK by cryotherapy and treatment with topical agents, such as fluorouracil, is among the most common causes of visits of elderly patients to dermatologists. AK and cSCC have long been known to be caused by UVB, and they have a high tumor mutation burden, with the mutation of p53 being among the most common. A recent extensive study by Thomson et al. [11] demonstrated additional mutations in tumor suppressor genes, such as Notch1, Notch2, FAT1 KMTC2, and HMCN1. In this study, the only dominant oncogenic mutation was in the phisphoinositol-3-kinase p85B. Our group was the first group to demonstrate that phosphoinositol-3 kinase inhibition in vivo led to decreased tumor growth in angiosarcoma, a tumor also associated with impaired p53 function.

Of note, TGFBR2 is downregulated in the progression from normal skin to AK to cSCC. This may represent an adaptation to scalp stroma, which becomes more rich in TGFb with aging.

## 4. Melanoma

The scalp is not an uncommon site for melanoma. Melanomas of the scalp have a distinct mutational profile compared to melanomas in other sites. Braf mutation is one of the most common driver mutations in melanoma, but the scalp has a different profile of Braf mutations, with V600K mutations being more common than V600E mutations, and V600K mutations appear to be more associated with chronic skin damage than V600E mutations [12,13,14]. Melanomas also appear to be more common in hair-bearing areas than in NMSC of the scalp, indicating that immunosurveillance may play less of a role in scalp melanoma than in NMSC [1,2,15]. Finally, mutations in other drivers are seen in scalp melanomas, including Rac1 and GNA11 [1,16,17,18]. An exceptional response was observed in a scalp melanoma with metastases in a patient treated with surgical debulking, gentian violet, and imiquimod, with no recurrence for over 2 years until the patient died of congestive heart failure [19]. Scalp melanomas thus appear in two clinical scenarios: one of chronic sun exposure and hair loss and one of sun protection and the retention of hair, occurring at a younger age [1].

## 5. Angiosarcoma

Angiosarcoma is a rare malignancy of endothelial cells, and the scalp is the most common site of cutaneous angiosarcoma. This tumor has a high propensity for distant metastases, and the prognosis is poor. Complete excision is often difficult due to large primary size and skip areas. In the largest study of angiosarcoma performed to date, angiosarcoma of the scalp has a high tumor mutation burden and UV signature compared to other sites. The mutation of p53 has also been observed nearly universally in scalp angiosarcoma [20]. The high tumor mutation burden of scalp angiosarcoma has suggested a role of immunotherapy, and some exceptional responses have been observed [20,21,22].

## 6. Treatment of Scalp Malignancies

The treatments of skin cancers on the scalp vary, and the treatment of choice depends on a multitude of factors including the following, amongst others: cancer type, size, and stage and general health status. Treatments may be divided into surgical or non-surgical treatments. Surgical options are usually the treatment of choice, and these include the following: curettage and electrodessication, excisional surgery, and Mohs micrographic surgery (MMS). MMS is a surgical technique which allows for an examination of 100% of the resected margins and achieves high cure rates while at the same time minimizing the unnecessary sacrificing of normal tissue. As such, the technique has been accepted as the treatment of choice for the majority of skin cancers on the scalp. Non-surgical treatments are usually advocated when surgery is not feasible either due to characteristics of the tumor or underlying illness contraindicating surgery.

Non-surgical treatments include the following:

1. Radiation therapy utilizes external beam radiation in which high-energy beams target the cancerous area. Additionally, Brachytherapy is a form of radiation therapy in which a radioactive material is placed in proximity to a tumor. The disadvantage of radiation therapy is the lack of precise treatment of only the tumor itself with the indiscriminate treatment of healthy tissue as well. As such, this treatment is prescribed either in cases of inoperable tumors or as an adjuvant treatment following surgical removal. Other disadvantages include the logistics of multiple patient visits to complete a course of radiation therapy.
2. Cryosurgery—Freezing cancer cells with liquid nitrogen is a viable treatment for lesions deemed to be pre-cancerous or superficial tumors. Similar to radiation therapy, this treatment is non-precise and often times is not successful in the eradication of all tumor cells. However, it is useful as a palliative measure in frail patients who may be intolerant to more aggressive therapies. Damage is caused by ice crystals disrupting the cell membrane and intracellular organelles.
3. Topical chemotherapy—The most commonly used agent is 5-fluorouracil (5-U), which is a topical chemotherapy which enables the successful treatment of pre-cancerous or superficial tumors in selective cases. Additionally, topical treatment with imiquimod cream via stimulating the immune system is an option for pre-cancerous lesions. Fluorouracil targets dividing cells, while imiquimod is a TLR7 (toll-like receptor) agonist.
4. Targeted therapy, immunotherapy, and chemotherapy are utilized in advanced cases when the tumor has spread beyond the scalp but are generally not used for scalp limited disease.

The current gold standard for the overwhelming majority of tumors, especially basal cell and squamous cell carcinomas of the scalp, is surgical removal utilizing the Mohs micrographic technique. As stated previously, this technique enables the removal of the tissue and the immediate evaluation of the entirety of the surgical margins. This is in contrast to the standard excision technique where the tissue is processed and examined in what is commonly referred to in a “bread loafing” sectioning. As such, the tumor may be missed and lead to a false negative pathology result. Additionally, in general, precautionary safety margins are taken in the common excision of tumors, often leading to the unnecessary sacrificing of healthy tissue, leading to a more complex reconstructive closure. The Mohs technique allows for minimal resection borders to be taken and thus minimizes the extent of reconstruction. One has to keep in mind that often times, the lack of laxity in the scalp region behooves one to attempt to cause as small a defect as possible, and we are able to do so without taking larger than necessary borders. In addition, the more extensive the surgery, the greater the risks for increased post-surgical complications such as bleeding and infection with prolonged healing and increased scarring. Patient satisfaction is very high as both the removal of the tumor and reconstruction are all performed on the same day while determining the complete removal of skin cancer. A tumor being determined to be inoperable and a patient not being seen as a candidate for surgery due to underlying disease are common reasons to resort to non-surgical treatment.

## 7. The Metabolic Parameters of the Aging Scalp

A major feature of the aging scalp is the loss of hair, manifesting in baldness. Aging-associated alopecia is primarily a nonscarring alopecia, characterized by the miniaturization of hair follicles, often associated with some degree of fibrosis [3,7]. The loss of longer hair likely diminishes the tonic IRF-4-IL-17 axis of the skin, allowing for a decreased level of immunosurveillance of UV-mutated keratinocytes and melanocytes. Hair requires ATP to maintain its complex, differentiated structure, and this requires optimal metabolism in both hair follicle keratinocytes and stromal support cells. As the scalp ages, there is decreased respiratory capacity in both hair-generating cells and stroma, and the IRF-4-IL-17 axis is replaced with a TGF-beta low-Sirt3 axis [8]. Low-Sirt3 fibroblasts are associated with tumor stroma, and they may play a supporting role in tumor progression. The clinical observation of increased skin cancer prevalence with low hair density reflects aging metabolism. The takeaway lessons from the carcinogenesis of the scalp are the following. First, hair does not provide as good a barrier to UV mutagenesis as commonly assumed. Wearing hats or other protective measures may prevent scalp carcinogenesis. Second, hair likely provides an immunological barrier to the development of skin cancer on the scalp, and interventions that increase IL-17 may be immunopreventive of scalp skin cancers. Third, metabolic stromal aging, mediated in part by dihydrotestosterone-reactive oxygen signaling, modifies the scalp into a less supportive environment for hair. At the same time, an increased level of TGF-beta, which decreases the level of the mitochondrial enzyme Sirt3, provides a permissive stroma that allows for the progression of mutated keratinocytes and melanocytes to frank malignancy. Sirt3 has been shown to be protective of cochlear hair cells [5,6], and Sirt3 activators like honokiol and methyl-honokiol have been shown to be beneficial in murine models of alopecia [23,24]. Pharmacologic interventions that maintain hair may also be preventive of scalp carcinogenesis.

## 8. IRF4—The Locus Between Hair Growth and Inflammation

In previous studies, we have shown that normal skin has tonic IL-12-mediated immunosurveillance, and an intact barrier function leads to the tonic production of IL-12, which suppresses both Th2- and Th17-mediated inflammation. Clinical observations suggest that the presence of hair skews the epidermis to a slight IL-17 predominance, which results in an increased frequency of IL-17-mediated inflammatory disorders, such as psoriasis, hidradenitis suppurativa, and seborrheic dermatitis in hair-bearing areas [25,26,27].

**Figure 1:**
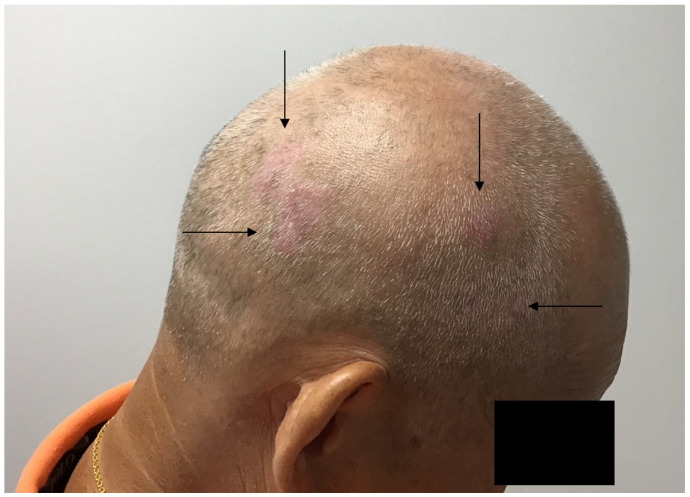
Psoriasis of the scalp is localized to the hair-bearing areas of the scalp (see arrows). Areas of pattern alopecia are spared from inflammation.

**Figure 2:**
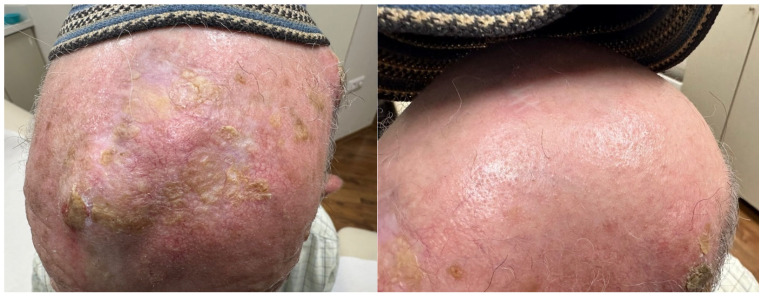
**Physical protection of scalp prevents carcinogenesis in areas of alopecia.** This patient with extensive solar damage has squamous cell carcinoma on scalp that was not protected by his headcovering (kipah) and clinically less solar damage on covered area. Kipahs are worn lifelong for males and thus may have prevented UV carcinogenesis.

IRF4 is a transcription factor that plays a role in pigmentation and IL-17-mediated inflammation [25,26,27,28,29]. IRF4 has been associated with several human pigmentation traits, including hair graying and hair loss, and IRF4 has also been shown to bind to the MITF (microphthalmia) promoter, which is the master transcriptional switch in melanocytes. The presence of IRF4 in hair may explain in part the presence of scalp psoriasis [25,26,27]. Interestingly, scalp inflammation is often localized to hair-bearing areas and is absent in areas of alopecia (Figure 1). Additional sun protective measures, such as the lifelong wearing of hats, may be protective of UV-induced carcinogenesis (Figure 2).

**Figure 3:**
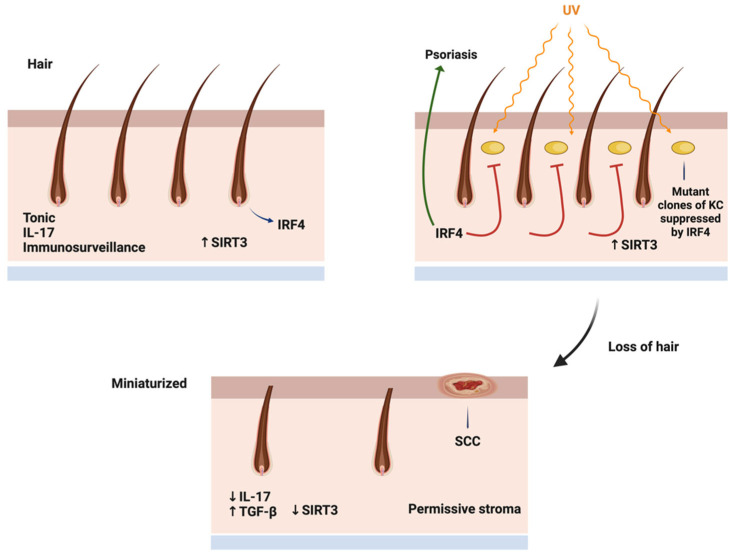
**Mechanism of immunometabolic regulation of scalp carcinogenesis.** In normal scalp, IRF4 transcription factor is highly expressed in hair follicles, and stroma promotes healthy hair. UV carcinogenesis successfully causes mutation in hair-bearing areas, but hair-derived IL-17 and high-Sirt3 stroma promote hair growth and impede tumor promotion. With hair loss, tonic IRF4/IL-17 production is lost, and stroma becomes elevated in TGF-beta and low in Sirt3, proving permissive environment for tumor progression.

Decreased IRF4 leads to hair graying, which in turn may lead to alopecia and the loss of immune protection against nonmelanoma skin cancer [3,7]. The loss of hair likely diminishes the presence of IL-17, resulting in the promotion of pre-existing UV mutant cells into clinically evident tumors (Figure 3).

## 9. IL-17, the Double-Edged Sword

We proposed that hair-derived IL-17 may play a protective role against the development of skin cancer. Others have shown that IL-17 can promote other cancers [30,31]. How do we reconcile these findings? A recent study demonstrated that the presence of wild-type p53 is required for the tumor-promoting activity of IL-17 [31]. We have previously shown that tumors with mutant p53 signal differently than tumors with wild-type p53. The majority of basal and squamous cell carcinomas have defects/mutations in p53, while the majority of melanomas have wild-type 53. This may explain in part why hair may be more protective against NMSC than melanoma.

## 10. Interplay of Genetics and Skin Carcinogenesis

AK are exceedingly common lesions on the scalp and a known precursor to cutaneous squamous cell carcinoma (cSCC). Genetic studies have implicated mutations in several genes in both AK and cSCC. These include mutations in p53, resulting in a gain of function, activating mutations in phosphoinositol-3 kinase p85, and inactivating mutations in Notch 1 and 2. Genetic predisposition to actinic keratoses has been linked to IRF4, a transcription factor which links immunity to pigmentation, as well as pigmentation-associated genes such as tyrosinase [25,26,27,28,29]. Current knowledge does not suggest a unique mutational profile of basal cell carcinoma of the scalp, with scalp and non-scalp basal cell carcinomas both having mutations in the Patch/Sonic hedgehog pathway.

## 11. Ultraviolet Light and Microbiome

As noted before, hair is an imperfect barrier to UV light. Parisi et al. calculated an ultraviolet protection factor (UPF) that ranged from approximately 5 to 17 in full sun, with the UPF increasing with higher solar zenith angles. Surprisingly, the UPF provided by shorter hair was generally higher by a range of 2–5 than that provided by longer hair—this was attributed to more parting of the subjects with long hair, which allowed for a greater surface area for UV exposure [32]. However, long hair may provide increased IL-17-mediated inflammation, which may balance out the increased UV exposure noted with long hair.

Additional factors that influence the UV penetration of the scalp include hair density, thickness, and color. De Gálvez et al. demonstrated that hair protects against both UVB and UVA radiation, and as expected, hair density, thickness, and the presence of melanin provide additional protection. White hair offers the least amount of protection [33]. Eyelashes also provide some degree of protection to the forehead, and similarly, Marro et al. found that light angle and the density of hair follicles influenced the degree of protection that hair provided [34].

Analyses of the scalp microbiome also provide hypotheses as to the contributory role of the scalp microbiome. The scalp microbiome is influenced by UV diminution, decreased pH, and moisture, which favors a different microbiome than sun-exposed skin. Lousada et al. noted that an increased abundance of both *Malassezia restricta* and *M. globose* has recently been reported in alopecic vertex areas compared with occipital skin and healthy controls [35]. Of interest, Malassezia globosa has been noted to have tumor-promoting activity and is found intratumorally in pancreatic cancer [36]. Malassezia is controlled by IL-17, and the loss of hair-derived IL-17 may allow for uncontrolled Malassezia growth and the promotion of existing mutations towards clinically evident cancer [37,38,39]. Thus, it is possible that Malassezia species may play a role in scalp carcinogenesis as well. The antifungal treatment of Malassezia should also be considered as a potential chemopreventive measure for scalp carcinogenesis.

Hair and nails are known for their production of antimicrobial peptides [40]. It is well known that nail dermatophytosis occurs more commonly in the elderly, and perhaps increased Malassezia colonization occurs in the elderly scalp. While it has not been well studied, the aging-related loss of antimicrobial peptides may be dependent on keratinocytes having a sufficient mitochondrial reserve to produce these peptides, which are not essential to the survival of keratinocytes.

## 12. Conclusions

The scalp is beginning to be appreciated as a distinct immunometabolic site. A novel candidate for the treatment of alopecia, a small-molecule mitochondrial pyruvate transport inhibitor, promotes hair growth in mice and is currently being evaluated in human clinical trials (**NCT06393452**). The proposed mechanism of action of this compound is the activation of lactate dehydrogenase (LDH), which activates hair stem cell growth. Of interest, Sirt3 also activates LDH by deacetylation. It is not known whether the activity of mitochondrial pyruvate transport inhibitors are Sirt3-dependent or -independent [41,42].

Finally, the understanding of scalp carcinogenesis has public health implications. It is generally assumed that the presence of hair is protective against carcinogenesis, and therefore, additional measures for sun protection are not needed. Clinical and scientific evidence suggests that hair does not prevent UV-induced mutations but may impede tumor progression until hair is lost. The presence of hair provides tonic IL-17 immunosurveillance and a stroma which may restrain tumor progression. Thus, additional measures to protect the scalp, i.e., hats, and agents that prevent hair loss may be preventive of future scalp carcinogenesis.

## Abbreviations

AK	actinic keratosis
cSCC	cutaneous squamous cell carcinoma
DP	dermal papilla
HFSC	hair follicle stem cell
HS	hair shaft
LDH	lactate dehydrogenase
MITF	microphthalmia
NMSC	nonmelanoma skin cancer
UPF	ultraviolet protection factor
